# Cryptic diversity in an Atlantic Forest malaria vector from the mountains of South-East Brazil

**DOI:** 10.1186/s13071-018-2615-0

**Published:** 2018-01-15

**Authors:** Guilherme de Rezende Dias, Thais Tenorio Soares Fujii, Bernardo Fernandes Fogel, Ricardo Lourenço-de-Oliveira, Teresa Fernandes Silva-do-Nascimento, André Nóbrega Pitaluga, Carlos José Carvalho-Pinto, Antonio Bernardo Carvalho, Alexandre Afrânio Peixoto, Luísa Damazio Pitaluga Rona

**Affiliations:** 1 Universidade Federal do Rio de Janeiro, IB, PPGBBE, Rio de Janeiro, RJ Brazil; 2 Universidade Federal do Rio de Janeiro, Polo de Xerém, Duque de Caxias, RJ Brazil; 3 Laboratório de Biologia Molecular de Insetos, IOC, FIOCRUZ, Rio de Janeiro, RJ Brazil; 4 Laboratório de Mosquitos Transmissores de Hematozoários, IOC, FIOCRUZ, Rio de Janeiro, RJ Brazil; 5 Laboratório de Biologia Molecular de Parasitas e Vetores, IOC, FIOCRUZ, Rio de Janeiro, RJ Brazil; 6 Universidade Federal de Santa Catarina, MIP, CCB, Florianópolis, SC Brazil; 7 Departamento de Genética, Universidade Federal do Rio de Janeiro, Rio de Janeiro, RJ Brazil; 8 Department of Life Sciences, Imperial College London, London, UK; 9 Universidade Federal de Santa Catarina, BEG, CCB, Florianópolis, SC Brazil; 10 Instituto Nacional de Ciência e Tecnologia em Entomologia Molecular (INCT-EM, CNPq), Rio de Janeiro, RJ Brazil

**Keywords:** *Anopheles* (*Kerteszia*) *cruzii*, Population genetics, Mosquitoes, Cryptic species, Speciation, Malaria

## Abstract

**Background:**

*Anopheles* (*Kerteszia*) *cruzii* is the primary vector of human and simian malarias in Brazilian regions covered by the Atlantic Rainforest. Previous studies found that *An. cruzii* presents high levels of behavioural, chromosomal and molecular polymorphisms, which led to the hypothesis that it may be a complex of cryptic species. Here, *An. cruzii* specimens were collected in five sites in South-East Brazil located at different altitudes on the inner and coastal slopes of two mountain ranges covered by Atlantic Rainforest, known as Serra do Mar and Serra da Mantiqueria. Partial sequences for two genes (*Clock* and *cpr*) were generated and compared with previously published sequences from Florianópolis (southern Brazil). Genetic diversity was analysed with estimates of population structure (*F*_*ST*_) and haplotype phylogenetic trees in order to understand how many species of the complex may occur in this biome and how populations across the species distribution are related.

**Results:**

The sequences from specimens collected at sites located on the lower coastal slopes of Serra do Mar (Guapimirim, Tinguá and Sana) clustered together in the phylogenetic analysis, while the major haplotypes from sites located on higher altitude and at the continental side of the same mountains (Bocaina) clustered with those from Serra da Mantiqueira (Itatiaia), an inner mountain range. These two *An. cruzii* lineages showed statistically significant genetic differentiation and fixed characters, and have high *F*_*ST*_ values typical of between species comparisons. Finally, in Bocaina, where the two lineages occur in sympatry, we found deviations from Hardy-Weinberg equilibrium due to a deficit of heterozygotes, indicating partial reproductive isolation. These results strongly suggest that at least two distinct lineages of *An. cruzii* (provisorily named “Group 1” and “Group 2”) occur in the mountains of South-East Brazil.

**Conclusions:**

At least two genetically distinct *An. cruzii* lineages occur in the Atlantic Forest covered mountains of South-East Brazil. The co-occurrence of distinct lineages of *An. cruzii* (possibly incipient species) in those mountains is an interesting biological phenomenon and may have important implications for malaria prevalence, *Plasmodium* transmission dynamics and control.

**Electronic supplementary material:**

The online version of this article (10.1186/s13071-018-2615-0) contains supplementary material, which is available to authorized users.

## Background

Autochthonous malaria transmission in Brazil is currently reported outside the Amazon region in areas covered by the Atlantic Rainforest, where vectors, humans and non-human primates co-exist [1, 2]. In this biome, one of world’s top biodiversity hotspots [3–6], the main vectors are *Anopheles* Meigen, 1818 mosquitoes of the subgenus *Kerteszia* Theobald, 1905, which uses *Bromeliaceae* water-tank plants as breeding sites [2, 7, 8]. *Anopheles* (*Kerteszia*) *cruzii* Dyar & Knab, 1908, known for its role as vector of the “bromeliad malaria” since the early twentieth century [9, 10], is considered the primary vector of human and simian malaria currently occurring in South (S) and South-East (SE) Brazilian regions [2, 11].

In the last 50 years several studies documented that *An. cruzii* populations from different regions of the country show great behavioural [12], morphological [8, 13], chromosomal [14–17] and molecular [18–24] polymorphism, leading to the hypothesis of *An. cruzii* being a complex of cryptic species. In Rio de Janeiro (RJ), Brazil’s third most densely populated state with 16 million habitants [25], more than 1700 malaria cases were reported from 1990 to 2015, mostly due to transmission by *An. cruzii* [1]. However, in spite of *An. cruzii* historical importance as a malaria vector and the continuous occurrence of autochthonous malaria cases in RJ, few studies have investigated the genetic diversity of this species in the state [2]. Research to date has tended to focus on comparing individuals from RJ mountains with those from other Brazilian states rather than investigating the genetic diversity among distinct sites within the state. A previous study using allozymes showed that a population from Nova Iguaçu, located near Tinguá, RJ, on the coastal side of Serra do Mar (a mountain range running parallel to the Atlantic Ocean from RJ to the South of Brazil) is genetically similar to the Florianópolis population, in the South Brazilian region; these localities are ~ 800 km apart [18].

**Fig. 1:**
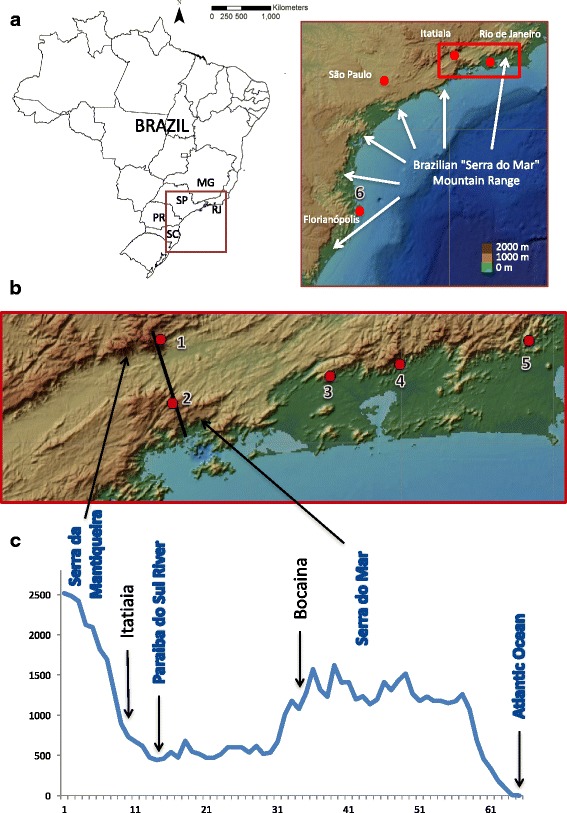
*Anopheles cruzii* collection sites and altitude profile in South-East Brazilian Atlantic Rainforest. **a** Map of Brazil showing the extension of the Serra do Mar mountain range throughout the South/South-East Brazillian coastline. **b** Magnification of the red box from (**a**) displaying the Paraiba Valley between Serra da Mantiqueira and Serra do Mar. The black line in (**b**) shows the terrain altitude profile (**c**) crossing from the Serra da Mantiqueira to the Atlantic Ocean. The collection sites are labelled as follows: 1, Itatiaia; 2, Bocaina; 3, Tinguá; 4, Guapimirim; 5, Sana; 6, Florianópolis. Sequences from Florianópolis (6), located in the Brazilian South Region, were retrieved from GenBank and included in the analysis for comparison. The altitude profile points are 1.25 km from each other and displays a horizontal scale of 1:500,000 and vertical scale of 1:50,000. (Source: Instituto Brasileiro de Geografia e Estatística - IBGE & NOAA)

On the other hand, multilocus DNA sequencing studies found evidence for two sympatric independent lineages, both of them distinct from lineages of other Brazilian states, occurring in Itatiaia, a site in the Serra da Mantiqueira mountains, which is located in the inner North-West of the state, separated from Serra do Mar by the Paraíba Valley [22, 24] (Fig. 1). However, since these studies were conducted separately and under different methodologies, the relationship between Serra da Mantiqueira and Serra do Mar populations remain unclear. Moreover, data from RJ are restricted to a small range of the *An. cruzii* distribution in the state and no further investigations have been made to understand the genetic diversity of the complex in this region.

The co-occurrence of more than one lineage of *An. cruzii* in the mountains of South-East Brazil may have important implications for malaria control, since they may be differentially involved in malaria transmission. Thus, three questions remain unsolved: (i) How many evolutionarily independent lineages of *An. cruzii* exists in the mountains of South-East Brazil and where do they occur? (ii) What is the relationship between *An. cruzii* individuals from distinct sites within the biome? (iii) If more than one lineage occur in South-East Brazil, which processes may have played a role in their differentiation? In order to answer these questions, this work analysed the genetic diversity of *An. cruzii* individuals from four sites covering the distribution of the Atlantic Forest throughout RJ, and one site in São Paulo’s Serra da Bocaina, and compared the genetic variability of these five populations from South-East Brazil to previously published data from Florianópolis (southern Brazil) [22, 24].

Partial fragments of *cpr* (211 bp) and *Clock* genes (224 bp) were used in this study, since they have previously been shown to be good markers for identification of differentiating lineages in *An. cruzii* [21, 22, 24] and also in the *Anopheles triannulatus* [26] complexes of cryptic species. The *cpr* gene (NADPH cytochrome P450 reductase), has a putative function in *Drosophila* odorant clearance [27]. Since olfactory cues may have important influence in mate recognition [28], *cpr* may be important in sexual isolation between closely related species. The *Clock* gene encodes a transcription factor important in the control of dipteran circadian cycles [29]. Circadian cycle genes have been useful in the identification of cryptic diversity in African anophelines [30] and other dipteran groups such as fruit flies [31] and sand flies [32]. Furthermore, since variability in daily rhythms have an influence on malaria transmission by anophelines [33], knowledge regarding divergence in this kind of molecular marker may lend to useful insights in to malaria dynamics of the Atlantic Forest.

Our results with these two markers (*cpr* and *Clock*) strongly suggest that there are at least two independent lineages of *An. cruzii* in the mountains of South-East Brazil.

## Methods

### Sampling sites, mosquito collection and morphological identification

Five sites in the Atlantic Forest areas of RJ and São Paulo (SP) States (both in South-East Brazil) were sampled between 2012 and 2013 (Fig. 1). Four of them are located in the Serra do Mar mountains, which extend for 1500 km from the South to South-East regions along the Brazilian coast: Bocaina (23°1'26.87"S, 44°43'6.47"W), Tinguá (22°35'31.30"S, 43°26'8.27"W), Guapimirim (22°30'30.72"S, 43°0'36.30"W) and Sana (22°24'0.00"S, 42°10'60.00"W). The fifth collection site was Itatiaia (22°27'46.74"S, 44°35'33.55"W), located at 900 m above sea level in Serra da Mantiqueira, an inner mountain range extending through SP, RJ and Minas Gerais States. Serra do Mar and Serra da Mantiqueira are separated by the Paraíba river valley. Bocaina is located on a plateau on the continental side of the Serra do Mar 1500 m above sea level, while Guapimirim, Sana and Tinguá are located on the coastal slopes of Serra do Mar below 600 m.

Adults and immatures of *An. cruzii* were collected as follows. The water collected from each bromeliad was kept in separate recipients and taken to the laboratory until the adults emerged. Additionally, adult mosquitoes were collected starting an hour before sunset and ending 2 hours after dusk (17:00 h to 20:00 h). Species identification was carried out using morphological identification keys [34]. Briefly, the morphological characters used to diagnose *An. cruzii* adults were: scutum with four dark longitudinal lines, fifth hind tarsomeres dark scaled on at least 50% at base, with an apical white band, wing vein R 4 + 5 white-scaled except for a small dark spot at the base and another at the apex. After identification, adult mosquitoes were kept in 95% ethanol at -20 °C for DNA extraction. The data for source (adult or reared in the laboratory) and sex of each sample are shown in Additional file 1: Table S1.

### Molecular analysis

The *cpr* and *Clock* partial gene sequences from Florianópolis and some sequences from Itatiaia (*cpr* gene: Ita02 to Ita12; *Clock* gene: Ita02 to Ita13) have already been published (GenBank accession numbers GU072619–GU072646 and GU072709–GU072730 for *cpr*; JX129234–JX129257 and GU016402–GU016425 for *Clock*; see Additional file 2 for details). These sequences were added to the study dataset in order to better understand how populations in RJ are related to those from other Brazilian sites which previously showed divergence when compared to Itatiaia [21–24]. The additional sequences from Itatiaia (10 mosquitoes for *cpr* and 8 for *Clock*) as well as those from Tinguá (10 mosquitoes for both genes), Guapimirim (16 mosquitoes for *cpr* and 9 for *Clock*), Sana (12 mosquitoes for *cpr* and 9 for *Clock*) and Bocaina (12 mosquitoes for *cpr* and 16 for *Clock*) were obtained by PCR, cloning and sequencing as described below. An alignment of all sequences for each gene is presented in Additional file 2.

Genomic DNA was extracted from each individual mosquito according to Jowett [35] and used in PCR reactions carried out in an Eppendorf Mastercycler® thermocycler (Westbury, USA) using the proofreading *Pfu* DNA polymerase (Biotools, Madrid, Spain) and previously described primers [21, 24]. PCR products were purified and cloned using CloneJET™ PCR Cloning Kit (Fermentas Life Sciences, Carlsbad, USA). Sequencing of positive clones was carried out in an ABI Prism 3730 DNA sequencer at the Oswaldo Cruz Institute using the ABI Prism Big Dye Terminator Cycle Sequencing Ready Reaction kit (Applied Biosystems, Foster City, USA). The identity of the cloned fragments was determined by BlastX analysis against the NCBI nr database (http://www.ncbi.nlm.nih.gov/BLAST/). At least 12 clones were sequenced for each mosquito to mitigate PCR errors, and allow the identification of the two alleles. The sequences were then edited and consensus sequences representing the two alleles were generated. The individuals were classified as homozygotes when only one haplotype was observed among the twelve sequences. The sequences were submitted to the GenBank database under the accession numbers KT724974–KT725197.

### DNA sequence analysis and haplotype genealogies

DNA sequences were aligned with ClustalX [36] and phylogenetic trees were constructed for each gene under the Bayesian Inference method using MrBayes v.3.2.5 [37, 38]. The best-fit substitution models F81 + G (*Clock* gene) and SYM + G (*cpr* gene) were selected following the BIC criterion as implemented in JModelTest [39]. Indel information was included in the analysis after coding gaps into a binary matrix using FastGap software [40]. Analysis ran for 5,000,000 generations (MCMC - 'burn-in' = 25%) from two independent random trees to generate a convergence diagnostic. The final trees presented here are the consensus between the two final trees generated for each gene. Results from all phylogenetic analyses were viewed in FigTree v.1.4.2 [41]. P_RO_S_EQ_ v.2.91 [42] and Arlequin v.3.11 [43] software were used to obtain pairwise estimates of population differentiation [44]. The comparison of genetic distances, estimated by pairwise *F*_*ST*_, with geographical distances by Mantel test [45] was performed with the *ade4* package [46] using R Software v.3.3.2 [47].

## Results

A total of 152 sequences (two alleles from each individual) were analysed for the *Clock* gene (32 sequences from Bocaina, 18 from Guapimirim, 40 from Itatiaia, 18 from Sana, 20 from Tinguá and 24 from Florianópolis). For the *cpr* gene, a total of 170 sequences were obtained (again the two alleles from each mosquito; 24 sequences from Bocaina, 32 from Guapimirim, 42 from Itatiaia, 24 from Sana, 20 from Tinguá and 28 from Florianópolis). Sequences from the *cpr* gene were 211 bp long and sequences from *Clock* were 224 bp long. Both loci include one intron that show a number of indels. All base substitutions were silent or occurred within the introns. An alignment of all sequences for each gene is presented in Additional file 2.

### Population structure

Table 1 shows the pairwise *F*_ST_ estimates for *Clock* and *cpr* genes. The highest *F*_ST_ values, all of them statistically significant (*P* < 0.005), were found in the comparisons between Itatiaia and Bocaina, with the other four populations from Florianópolis, Guapimirim, Tinguá and Sana (*F*_ST_ between 0.423–0.734 for *Clock* and between 0.388–0.733 for *cpr*). Such differentiation is unexpected within a single evolutionary lineage: it has been proposed that *F*_*ST*_ values above 0.35 correspond to different species [48]. On the other hand, all comparisons between Bocaina and Itatiaia, or between the four other populations yield much lower *F*_ST_ values (all below 0.35). These results suggest that the sampled populations belong to at least two independent evolutionary lineages, i.e. to different incipient species.

**Table 1:** Genetic and geographical distances between *Anopheles cruzii* populations. Pairwise *F*_*ST*_ (estimates of population differentiation) and *P*-values (significance of *F*_*ST*_ values evaluated by 1000 random permutations) were calculated between sequences from individuals of distinct localities

Population/Gene	*Clock*		*cpr*		Distance (km)^a^
	*F*_*ST*_	*P-*value	*F*_*ST*_	*P-*value	
Guapimirim *vs* Florianópolis	0.001	0.315	0.244	< 0.001	785
Tinguá *vs* Guapimirim	0.004	0.346	0.035	0.092	45
Tinguá *vs* Florianópolis	0.008	0.320	0.322	< 0.001	748
Tinguá *vs* Sana	0.012	0.270	0.160	0.009	130
Guapimirim *vs* Sana	0.063	0.073	0.124	< 0.001	86
Sana *vs* Florianópolis	0.083	0.009	0.298	< 0.001	854
Bocaina *vs* Itatiaia	0.013	0.089	0.273	< 0.001	64
Bocaina *vs* Guapimirim	0.423	< 0.001	0.395	< 0.001	184
Bocaina *vs* Tinguá	0.429	< 0.001	0.446	< 0.001	140
Bocaina *vs* Sana	0.454	< 0.001	0.388	< 0.001	269
Bocaina *vs* Florianópolis	0.470	< 0.001	0.516	< 0.001	628
Itatiaia *vs* Guapimirim	0.673	< 0.001	0.643	< 0.001	163
Itatiaia *vs* Tinguá	0.676	< 0.001	0.709	< 0.001	120
Itatiaia *vs* Sana	0.722	< 0.001	0.652	< 0.001	248
Itatiaia *vs* Florianópolis	0.734	< 0.001	0.733	< 0.001	686

^a^The approximate geographical distances between localities in km

It is interesting to examine in more detail the *F*_ST_ data. The *Clock* pairwise *F*_ST_ values comparing Itatiaia with Bocaina and also those values comparing the populations from Group 1 were usually under 0.08 and most of them were not statistically significant. Using the *cpr* gene, albeit most of them significant, the pairwise *F*_ST_ values among Guapimirim, Tinguá and Sana (from Group 1) were low, usually under 0.16, while those between Florianópolis and these other three populations from Group 1 were moderately high (from 0.24 to 0.32). This differentiation of the Florianópolis population is somewhat expected, given that it is located ~ 800 km away from the remaining ones, whereas Guapimirim, Tinguá and Sana are at most 130 km apart. However, the differentiation among *An. cruzii* populations cannot be explained by a simple isolation by distance model: the correlation between pairwise *F*_ST_ values and geographical distance was low and statistically non-significant for both genes (*cpr*: *r* = 0.127, *P* = 0.22; *Clock*: *r* = -0.097, *P* = 0.46).

### Haplotype genealogies

**Fig. 2:**
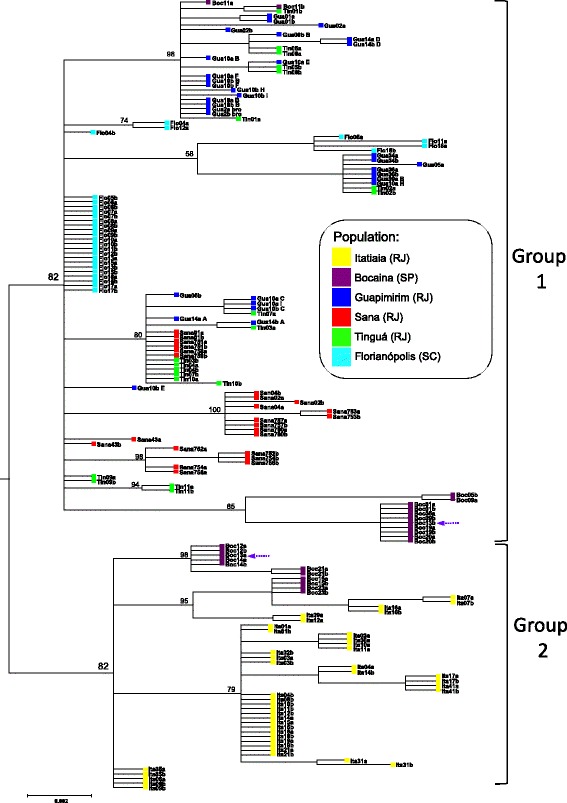
Bayesian inference analysis tree based on *cpr* sequences (SYM + G model). Genealogical relationships of the haplotypes show that at least two distinct *Anopheles cruzii* groups (1 and 2) occur in Rio de Janeiro State. Posterior probability values are represented above nodes. Lowercase letters in the haplotype names specify the two alleles (*a* or *b*) obtained from each individual. Purple dashed arrows indicate haplotypes from distinct groups (1 or 2) occurring in the same individual (GenBank accession numbers for all samples of *cpr* tree: KT724974–T725093, GU072619–GU072646 and GU072709–GU072730)

**Fig. 3:**
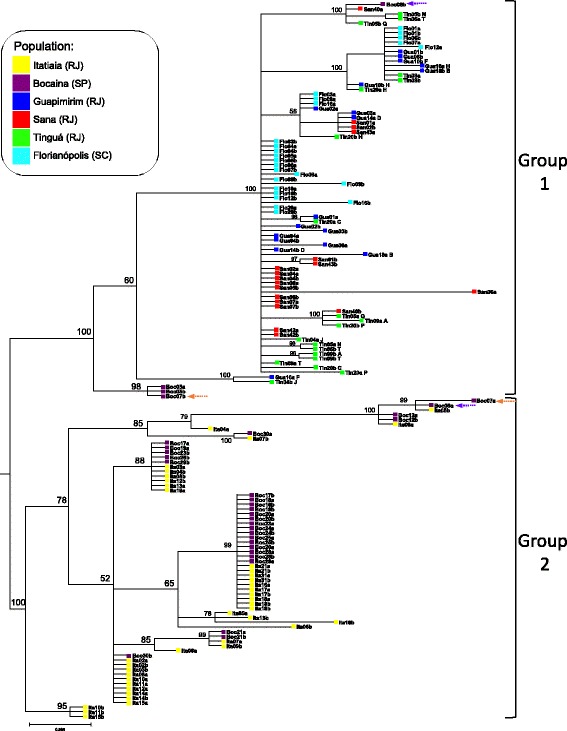
Bayesian inference analysis tree based on *Clock* sequences (F81 + G model). Genealogical relationships of the haplotypes show that at least two distinct *Anopheles cruzii* groups (1 and 2) occur in Rio de Janeiro State. Posterior probability values are represented above nodes. Lowercase letters in the haplotype names specify the two alleles (*a* or *b*) obtained from each individual. Purple and orange dashed arrows indicate haplotypes from distinct groups (1 or 2) occurring in the same individual (GenBank accession numbers for all samples of *Clock* tree: KT725094–KT725197, JX129234–JX129257 and GU016402–GU016425)

In order to gain a deeper insight on the differentiation of the *An. cruzii* populations, phylogenetic trees of the alleles were generated under the Bayesian Inference method for the two genes, *cpr* and *Clock* (Figs. 2 and 3). In both trees, the most basal split separated the *An. cruzii* haplotypes in two large groups (henceforth called Groups 1 and 2) with posterior probabilities (pp) of 82% for *cpr* and 100% for *Clock*.

Group 1 was composed mainly of sequences obtained from individuals from the coastal side of Serra do Mar mountain range (Sana, Guapimirim and Tinguá) and was the same occurring in Florianópolis (South Brazil). Group 2 was composed exclusively by sequences from Itatiaia and Bocaina individuals in both trees. Hence the phylogenetic trees agree with the *F*_ST_ data analysed in the previous section, i.e. the sampled *An. cruzii* populations belong to at least two incipient species (Group 1 and Group 2) that cannot be distinguished morphologically, at least using the available taxonomic keys.

### Sympatry of group 1 and group 2 haplotypes in the Bocaina population

The phylogenetic analyses disclosed another interesting phenomenon: the Bocaina population, which was sampled for the first time in the present study, contained Group 1 and Group 2 haplotypes for both genes (Figs. 2 and 3), i.e. the incipient species would be sympatric there. This allows an additional test for the hypothesis that Group 1 and Group 2 correspond to different species: if this is true, it is possible (although not mandatory) that we will find deviations from the Hardy-Weinberg equilibrium (namely, reduced amount of heterozygotes) due to partial reproductive isolation. Indeed, we observed 6 individuals *cpr*_1_/*cpr*_1_, 5 *cpr*_2_/*cpr*_2_ and 1 *cpr*_1_/*cpr*_2_, whereas the expected counts are 3.5, 2.5 and 6, respectively (*χ*^2^ = 8.4, *df* = 1, *P* = 0.004). For the *Clock* gene, the heterozygote deficiency is less pronounced and not statistically significant (observed: 1 *Clock*_1_/*Clock*_1_, 13 *Clock*_2_/*Clock*_2_ and 2 *Clock*_1_/*Clock*_2_; expected: 0.25 *Clock*_1_/*Clock*_1_, 12.25 *Clock*_2_/*Clock*_2_ and 3.5 *Clock*_1_/*Clock*_2_, respectively; *P* > 0.05). The most probable explanation for the deficiency of heterozygotes in sympatry is reproductive isolation [49].

It is important to note that the Bocaina population does not represent a simple scenario of two completely isolated species: we observed one *cpr*_1_/*cpr*_2_ heterozygote (individual Boc13, which would be a hybrid; marked with a purple dashed arrow in Fig. 2), and two *Clock*_1_/*Clock*_2_ heterozygotes (Boc07 and Boc08; purple and orange dashed arrows in Fig. 3). The overall frequency of hybrids would be around 10% (1 out 12 for *cpr*; 2 out 16 for *Clock*). Furthermore, the *Clock* and *cpr* genotypes are not always coherent in Bocaina individuals (namely, individuals 19 and 20 are *cpr*_1_/*cpr*_1_ and *Clock*_2_/*Clock*_2_), which is compatible with introgression or incomplete lineage sorting [49]. This complex scenario of partial isolation and differential introgression is similar to what has been observed in other anopheline species [50, 51]. Further studies of *An. cruzii* populations, particularly focusing in Bocaina and using a broader range of markers, are necessary to better understand the phenomenon.

### Fixed differences in *cpr* sequences

All variations in the *cpr* gene occurred in the intron, which shows a number of polymorphic indels and microsatellites (Additional file 2). The analysis of these polymorphisms showed that fixed differences are present between individuals from Groups 1 and 2. Table 2 shows the number of copies of each repeat in *An. cruzii* studied populations. All individuals from Itatiaia have AAA at position 18, whereas all individuals from the four populations from Group 1 have AA. In Bocaina, both repeat patterns occur: Bocaina sequences from Group 2 are AAA and those from Group 1 are AA. Individuals from Florianópolis, Sana, Guapimirim and Tinguá have two to three “CG” repeats at position 21 and six to nine “CT” repeats at position 147, whereas in Itatiaia all individuals have just one “CG” and four “CT” repeats. Once again Bocaina looks somewhat intermediate: individuals from there have one or two “CG” repeats and four to seven “CT” repeats. Fixed differences data are summarised in Table 2.

**Table 2:** Microsatellite repeat polymorphisms in *cpr* sequences from individuals of distinct localities reveals fixed differences. Repeat polymorphisms were identified in the *cpr* sequences from individuals sampled in distinct localities. Itatiaia sequences have fixed differences [(A)_3_, (CG)_1_, (CT)_4_] when compared with those from Florianópolis, Guapimirim, Tinguá and Sana [(A)_2_, (CG)_2–3_, (CT)_6–9_]. In Bocaina, both repeat patterns co-occur. Based on Additional file 2, (A)_2–3_ repeats are located between positions 18 to 20, (CG)_1–3_ between 21 to 26 and (CT)_4–9_ between 147 to 164 positions

Locality	Number of repeats		
	A	CG	CT
Florianópolis	2	2	6–9
Guapimirim	2	2	6–8
Tinguá	2	2	6–8
Sana	2	2–3	7–8
Bocaina	2–3	1–2	4–7
Itatiaia	3	1	4

## Discussion

Several studies have shown significant differentiation among *An. cruzii* populations [8, 12–24]. We found that at least two independent *An. cruzii* groups (probably incipient species) occur in South and South-East Brazillian Atlantic Rainforests: Group 1 encompasses populations from Florianópolis, Guapimirim, Tinguá and Sana as well as part of the Bocaina sample, and Group 2 is composed of Itatiaia and the remainder of the Bocaina sample. There is good evidence that the distinction between Group 1 and Group 2 is real and biologically meaningful: (i) it is the deepest node in the haplotype phylogeny of both *cpr* and *Clock* genes, with strong statistical support (pp of 82 and 100%, respectively); (ii) there are fixed differences between them (Table 2); (iii) Group 1 and Group 2 occur in sympatry in Bocaina and we found a statistically significant deficiency in heterozygote frequency for the *cpr* gene, indicating partial reproductive isolation; (iv) the genetic divergence between Group 1 and Group 2 populations is very high, with an average *F*_*ST*_ of 0.57 (Table 1). Such differentiation is greater than expected within a single evolutionary lineage without any kind of reproductive isolation; *F*_*ST*_ values above 0.35 usually correspond to different species [48]. Our data reject the scenario of a single species with broad distribution in which physically distant populations are more differentiated (i.e. a simple isolation by distance model). For example, Tinguá is 120 km apart from Itatiaia and 750 km apart from Florianópolis, and yet the population from this locality is genetically much more similar to Florianópolis (Table 1); overall, there was no significant correlation between geographical and genetic distance (*r* = 0.127 and -0.097 for *cpr* and *Clock*, respectively; Table 1). Hence, it seems safe to conclude that there are at least two different species in the sampled populations of *An. cruzii*. They are probably incipient species, since we observed Group 1 / Group 2 hybrids in the Bocaina population, where the two forms occur in sympatry. Overall, the *An. cruzii* case seems similar to that of the *An. gambiae* complex [51]: a complex of closely related species with partial introgression, which originates a patchwork of undifferentiated and differentiated genomic regions, with some markers showing more clear evidences of reproductive isolation (e.g. rDNA “M” and “S” forms in *An. gambiae*; *cpr* “Group 1” and “Group 2” alleles in *An. cruzii*).

Further studies of *An. cruzii* populations, particularly focusing in Bocaina and using a broader range of markers, are clearly desirable, since there are many interesting open questions. For example, what is the introgression pattern across the *An. cruzii* genome? Are there additional species? A possible hint for this latter possibility is the observation of some differentiation within Group 1 haplotypes: the Bocaina sequences do not appear at random in *cpr* and *Clock* trees, but instead formed a well defined cluster with strong statistical support (Figs. 2 and 3). We are now pursuing these two questions.

The results presented here are consistent with previous studies. A study using allozymes as genetic markers concluded that no significant differences could be found between *An. cruzii* populations from Florianopolis (SC) and Tinguá (RJ) [18]. On the other hand, sequences from Itatiaia (SE) were distinct from those from Florianópolis (S) [24], which we confirmed here with a larger sample.

A question that warrants future studies is: what is the cause and history of the differentiation between Group 1 and Group 2 populations? Geographical distance per se is not a likely explanation. These mosquitoes strictly depend on bromeliad for their life-cycle, and the rainforests at the top of mountain ranges were mostly replaced by grassland during pre- and full-glacial times [52]. Thus, repeated periods of isolation in the last 2.5 Myr between *An. cruzii* populations from the coastal slope of Serra do Mar (e.g. Tinguá) and Serra da Mantiqueira (e.g. Itatiaia) might have happened; such dynamics are known to have favoured speciation in groups as diverse as flatworms [53], harvestmen [54], snakes [55], frogs [56–60], primates [61–63], rodents [64], birds [65–67] and several malaria vectors [68]. Under this scenario, the Bocaina population, which is located midway between the coastal Serra do Mar and Serra da Mantiqueira populations, could be a zone of secondary contact between the incipient species, which typically is accompanied by hybridization and partial introgression [69, 70].

Another open question, this time with direct implications for human health, is: are there differences in the vectorial capacities of the distinct lineages of *An. cruzii*? To date, the autochthonous human malaria cases from RJ seem to be restricted to the coastal side of Serra do Mar mountains, where *An. cruzii* is considered the main vector [2, 71, 72]. So, the knowledge about the co-occurrence of more than one lineage of *An. cruzii* in those mountains may have important implications for malaria control, especially if they are differentially involved in malaria transmission.

## Conclusions

The analysis of genetic differentiation of *cpr* and *Clock* genes indicates that at least two evolutionarily independent lineages of *An. cruzii* (probably incipient species) occur in the Atlantic Forests of South-East Brazil. Individuals from these lineages present significant genetic divergence and fixed differences. One of these lineages, Group 1, occurs mainly on the coastal side of the Serra do Mar mountain range in altitudes below 600 m and Group 2 occurs in the inner sites of Serra do Mar and on the Serra da Mantiqueira mountains at higher altitudes (above 900 m). The two groups occur in sympatry in the Bocaina population, where we found evidence for incomplete reproductive isolation.

## Additional files

Additional file 1: Table S1. Source (adult/ bromeliad: reared in the laboratory) and sex of each sample used in this study. In Bocaina, captures of only adult females were performed; in Guapimirim and Sana, captures of only immatures were executed; and in Itatiaia and Tinguá, captures of both adults and immatures were carried out. ♂, males; ♀, females;?, undetermined sex. (DOCX 15 kb)

Additional file 2: DNA sequences alignments of the *cpr* and *Clock* gene fragments from all *Anopheles cruzii* populations analysed. The introns are presented in the darkened regions. Dots represent the identity of the first nucleotide sequence. *Abbreviations*: Flo, individuals from Florianópolis; Boc, Bocaina; Gua, Guapimirim; Ita, Itatiaia; San: Sana, Tin: Tinguá. (DOC 114 kb)
