# Supplementary material for: Cryptic diversity in an Atlantic Forest malaria vector from the mountains of South-East Brazil
Source: Parasit Vectors. 2018 Jan 15;11:36. doi: 10.1186/s13071-018-2615-0 (PMC5769553; doi:10.1186/s13071-018-2615-0)
Supplement: Supplementary file 1 — Source (adult/ bromeliad: reared in the laboratory) and sex of each sample used in this study. In Bocaina, captures of only adult females were performed; in Guapimirim and Sana, captures of only immatures were executed; and in Itatiaia and Tinguá, captures of both adults and immatures were carried out. ♂, males; ♀, females;?, undetermined sex. (DOCX 15 kb) [file 13071_2018_2615_MOESM1_ESM.docx]

**Additional file 1: Table S1.** Source (adult / bromeliad: reared in the laboratory) and sex of each sample used in this study. In Bocaina, captures of only adult females were performed; in Guapimirim and Sana, captures of only immatures were executed; and in Itatiaia and Tinguá, captures of both adults and immatures were carried out. ♂, males; ♀, females; ?, undetermined sex.

| Guapimirim | Sex | Sana | Sex | Itatiaia | Sex | Source | Tinguá | Sex | Source |
| --- | --- | --- | --- | --- | --- | --- | --- | --- | --- |
| 01 | ♂ | 01 | ♂ | 01 | ♂ | Bromeliad | 01 | ♀ | Adult |
| 02 | ♂ | 02 | ♀ | 14 | ♂ | Bromeliad | 02 | ♀ | Adult |
| 03 | ♂ | 04 | ♀ | 15 | ♂ | Bromeliad | 03 | ♀ | Adult |
| 04 | ♂ | 05 | ♀ | 16 | ♂ | Bromeliad | 04 | ? | Bromeliad |
| 05 | ♂ | 06 | ♀ | 17 | ♂ | Bromeliad | 05 | ? | Bromeliad |
| 06 | ♀ | 07 | ♂ | 18 | ♂ | Bromeliad | 06 | ? | Bromeliad |
| 09B | ♀ | 40 | ♀ | 19 | ♂ | Bromeliad | 07 | ? | Bromeliad |
| 10B | ♂ | 42 | ♀ | 21 | ♀ | Adult | 09 | ♀ | Bromeliad |
| 10C | ♂ | 43 | ♀ | 31 | ♀ | Adult | 10 | ? | Bromeliad |
| 10E | ♂ | 751 | ♂ | 41 | ♀ | Adult | 11 | ? | Bromeliad |
| 10F | ♂ | 752 | ♀ |  |  |  | 04J | ? | Bromeliad |
| 10H | ♀ | 753 | ♂ |  |  |  | 05N | ? | Bromeliad |
| 10I | ♀ | 754 | ♀ |  |  |  | 05Q | ? | Bromeliad |
| 14A | ♀ | 756 | ♀ |  |  |  | 05T | ? | Bromeliad |
| 14D | ♀ | 757 | ♂ |  |  |  | 09A | ? | Bromeliad |
| 18B | ♀ | 759 | ♀ |  |  |  | 09T | ? | Bromeliad |
| 2bro | ♀ | 760 | ♀ |  |  |  | 20C | ? | Bromeliad |
|  |  |  |  |  |  |  | 20H | ♂ | Bromeliad |
|  |  |  |  |  |  |  | 20P | ♂ | Bromeliad |
