# Supplementary material for: Cryptic diversity in an Atlantic Forest malaria vector from the mountains of South-East Brazil
Source: Parasit Vectors. 2018 Jan 15;11:36. doi: 10.1186/s13071-018-2615-0 (PMC5769553; doi:10.1186/s13071-018-2615-0)
Supplement: Supplementary file 2 — DNA sequences alignments of the cpr and Clock gene fragments from all Anopheles cruzii populations analysed. The introns are presented in the darkened regions. Dots represent the identity of the first nucleotide sequence. Abbreviations: Flo, individuals from Florianópolis; Boc, Bocaina; Gua, Guapimirim; Ita, Itatiaia; San: Sana, Tin: Tinguá. (DOC 114 kb) [file 13071_2018_2615_MOESM2_ESM.doc]

**Additional file 2:** DNA sequences alignments of the *cpr* and *Clock* gene fragments from all *Anopheles cruzii* populations analysed. The introns are presented in the darkened regions. Dots represent the identity of the first nucleotide sequence. *Abbreviations*: Flo, individuals from Florianópolis; Boc, Bocaina; Gua, Guapimirim; Ita, Itatiaia; San: Sana, Tin: Tinguá.

Alignment of the DNA sequences from the *cpr* gene fragment.

0000000000000000000000000000000000000000000000000000000000000000000000000000000000000000000000000001111111111111111111111111111111111111111111111111111111111111111111111111111111111111111111111111111222222222222

0000000001111111111222222222233333333334444444444555555555566666666667777777777888888888899999999990000000000111111111122222222223333333333444444444455555555556666666666777777777788888888889999999999000000000011

1234567890123456789012345678901234567890123456789012345678901234567890123456789012345678901234567890123456789012345678901234567890123456789012345678901234567890123456789012345678901234567890123456789012345678901

Boc01a GTGTAATATGGTAAGCGAA-CG----AGAGAGAG--------------------------------------------TCCTCGCCTATACGGTGACGCCGGCGGGCGCG--------CCAGCATGTTGTAATCCGTTCCGTTCCACTCTCTCTCTCTCT----GTGCACACGCAGGAAGAGCTGTTGCAGCTGAAAGACATCGAGAAATC

Boc01b ...................-..----........--------------------------------------------................................--------..........................................----...............................................

Boc05a ...................-..----........--------------------------------------------................................--------..........................................----...............................................

Boc05b ...................-..----........--------------------------------------------...G..A.....................------------........................................------...............................................

Boc09a ...................-..----........--------------------------------------------...G..A.....................------------........................................------...............................................

Boc09b ...................-..----........--------------------------------------------................................--------..........................................----...............................................

Boc11a ...................-..CG--........AGAGAGAGAGAGAGAGAGAGAGAG--------------------...G........T.................G.CGGG----..........................................----...............................................

Boc11b ...................-..CG--........AGAGAGAGAGAGAGAGAGAGAG----------------------...G........T.................G.CGGG----..........................................----...............................................

Boc12a ...................A..----....G...AGAG----------------------------------------...G..........................G.CGGG----....................................----------...............................................

Boc12b ...................A..----....G...AGAG----------------------------------------...G..........................G.CGGG----....................................----------...............................................

Boc13a ...................A..----....G...AGAG----------------------------------------...G..........................G.CGGG----....................................----------...............................................

Boc13b ...................-..----........--------------------------------------------................................--------..........................................----...............................................

Boc14a ...................A..----....G...AGAG----------------------------------------...G..........................G.CGGG----....................................----------...............................................

Boc14b ...................A..----....G...AGAG----------------------------------------...G..........................G.CGGG----....................................----------...............................................

Boc15a ...................A..----........AGAG----------------------------------------...G..........................G.CGAG----......................A.............----------...............................................

Boc15b ...................A..----........AGAG----------------------------------------...G..........................G.CGAG----......................A.............----------...............................................

Boc19a ...................-..----........--------------------------------------------................................--------..........................................----...............................................

Boc19b ...................-..----........--------------------------------------------................................--------..........................................----...............................................

Boc20a ...................-..----........--------------------------------------------................................--------..........................................----...............................................

Boc20b ...................-..----........--------------------------------------------................................--------..........................................----...............................................

Boc21a ...................A..----....G...AGAG----------------------------------------...G..........................G.TGGG----....................................----------...............................................

Boc21b ...................A..----....G...AGAG----------------------------------------...G..........................G.TGGG----....................................----------...............................................

Boc23a ...................A..----........AGAG----------------------------------------...G..........................G.CGAG----......................A.............----------...............................................

Boc23b ...................A..----........AGAG----------------------------------------...G..........................G.CGAG----......................A.............----------...............................................

Flo04a ...................-..CG--........AGAG----------------------------------------...G..........................G.CGGG----..........................................CTCT...............................................

Flo04b ...................-..CG--........AGAG----------------------------------------...G..........................G.CGGG----..........................................CT--...............................................

Flo05a ...................-..CG--........AGAGAGAGAGAGAG------------------------------...G..........................G-CGGGCGGG..........................................----...............................................

Flo05b ...................-..CG--........AGAG----------------------------------------...G..........................G.CGGG----..........................................CT--...............................................

Flo06a ...................-..CG--........AGAG----------------------------------------...G..........................G.CGGG----..........................................CT--...............................................

Flo06b ...................-..CG--........AGAG----------------------------------------...G..........................G.CGGG----..........................................CT--...............................................

Flo07a ...................-..CG--........AGAG----------------------------------------...G..........................G.CGGG----..........................................CT--...............................................

Flo07b ...................-..CG--........AGAG----------------------------------------...G..........................G.CGGG----..........................................CT--...............................................

Flo08a ...................-..CG--........AGAG----------------------------------------...G..........................G.CGGG----..........................................CT--...............................................

Flo08b ...................-..CG--........AGAG----------------------------------------...G..........................G.CGGG----..........................................----...............................................

Flo09a ...................-..CG--........AGAG----------------------------------------...G..........................G.CGGG----..........................................CT--...............................................

Flo09b ...................-..CG--........AGAG----------------------------------------...G..........................G.CGGG----..........................................CT--...............................................

Flo10a ...................-..CG--........AGAG----------------------------------------...G..........................G.CGGG----..........................................CT--...............................................

Flo10b ...................-..CG--........AGAG----------------------------------------...G..........................G.CGGG----..........................................CT--...............................................

Flo11a ...................-..CG--........AGAGAGAGAGAA--------------------------------...G..........................G-CGGGCGGG..........................................----...............................................

Flo11b ...................-..CG--........AGAG----------------------------------------...G..........................G.CGGG----..........................................CT--...............................................

Flo12a ...................-..CG--........AGAG----------------------------------------...G..........................G.CGGG----..........................................CTCT...............................................

Flo12b ...................-..CG--........AGAG----------------------------------------...G..........................G.CGGG----..........................................CT--...............................................

Flo13a ...................-..CG--........AGAG----------------------------------------...G..........................G.CGGG----..........................................CT--...............................................

Flo13b ...................-..CG--........AGAG----------------------------------------...G..........................G.CGGG----..........................................CT--...............................................

Flo15a ...................-..CG--........AGAG----------------------------------------...G..........................G.CGGG----..........................................CT--...............................................

Flo15b ...................-..CG--........AGAG----------------------------------------...G..........................G.CGGG----..........................................CT--...............................................

Flo16a ...................-..CG--........AGAG----------------------------------------...G..........................G.CGGG----..........................................CT--...............................................

Flo16b ...................-..CG--........AGAG----------------------------------------...G..........................G.CGGG----..........................................CT--...............................................

Flo17a ...................-..CG--........AGAG----------------------------------------...G..........................G.CGGG----..........................................CT--...............................................

Flo17b ...................-..CG--........AGAG----------------------------------------...G..........................G.CGGG----..........................................CT--...............................................

Flo18a ...................-..CG--........AGAGAGAGAGAGAA------------------------------...G..........................G-CGGGCGGG........................................------...............................................

Flo18b ...................-..CG--........AGAGAGAGAGAGAGAA----------------------------...G..........................G-CGGGCGGG..........................................----...............................................

Gua01a ...................-..CG--........AGAGAGAGAGAGAGAGAGAGAGAGAG------------------...G........T.................G.CGGG----..........................................----...............................................

Gua01b ...................-..CG--........AGAGAGAGAGAGAGAGAGAGAGAGAG------------------...G........T.................G.CGGG----..........................................----...............................................

Gua02a ...................-..CG--........AGAGAGAGAGAGAGAGAGAGAGAGAGAGAGAG------------...G........T.................G.CGGG----.....G.....................G..............----...............................................

Gua02b ...................-..CG--........AGAGAGAGAGAGAGAGAGAGAGAGAGAGAGAGAGAG--------...G........T.................G.CGGG----..........................................----...............................................

Gua04a ...................-..CG--........AGAG----------------------------------------...G..........................G-CGGGCGGG....................................T.....CT--...............................................

Gua04b ...................-..CG--........AGAG----------------------------------------...G..........................G-CGGGCGGG....................................T.....CT--...............................................

Gua05a ...................-..CG--........--------------------------------------------...G..........................G-CGGGCGGG....................................T.....CT--...............................................

Gua05b ...................-..CG--........AGAG----------------------------------------...G..........................GCCGGG----..........................................----...............................................

Gua06a ...................-..CG--........AGAG----------------------------------------...G..........................G-CGGGCGGG....................................T.....CT--...............................................

Gua06b ...................-..CG--........AGAG----------------------------------------...G..........................G-CGGGCGGG....................................T.....CT--...............................................

Gua09a_B ...................-..CG--........AGAG----------------------------------------...G..........................G-CGGGCGGG....................................T.....CT--...............................................

Gua09b_B ...................-..CG--........AGAGAGAGAGAGAGAGAGAG------------------------...G........T.................G.CGGG----..........................................----...............................................

Gua10a_B ...................-..CG--........AGAGAGAGAGAGAGAGAGAGAGAG--------------------...G........T.................G.CGGG----..........................................----...............................................

Gua10b_B ...................-..CG--........AGAGAGAGAGAGAGAGAGAGAGAG--------------------...G........T.................G.CGGG----..........................................----...............................................

Gua10a_C ...................-..CG--........AGAGAGAG------------------------------------...G..........................GCCGGG----..........................................----...............................................

Gua10b_C ...................-..CG--........AGAGAGAG------------------------------------...G..........................GCCGGG----..........................................----...............................................

Gua10a_E ...................-..CG--........AGAGAGAGAGAGAGAGAG--------------------------...G........T.................G.CGGG----..........................................----...............................................

Gua10b_E ...................-..CG--........AGAG----------------------------------------...G..........................G.CGGG----........................................------...............................................

Gua10a_F ...................-..CG--........AGAGAGAGAGAGAGAGAGAGAGAGAGAGAGAGAGAGAGAG----...G........T.................G.CGGG----..........................................----...............................................

Gua10b_F ...................-..CG--........AGAGAGAGAGAGAGAGAGAGAGAGAGAGAGAGAGAGAGAG----...G........T.................G.CGGG----..........................................----...............................................

Gua10a_H ...................-..CG--........AGAG----------------------------------------...G..........................G-CGGGCGGG....................................T.....CT--...............................................

Gua10b_H ...................-..CG--........AGAGAGAGAGAGAGAGAGAGAGAGAGAGAGAGAG----------...G........T.................G.CGGG----..........................................----...............................................

Gua10a_I ...................-..CG--........AGAGAGAG------------------------------------...G..........................GCCGGG----..........................................----...............................................

Gua10b_I ...................-..CG--........AGAGAGAGAGAGAGAG----------------------------...G........T.................G.CGGG----..........................................----...............................................

Gua14a_A ...................-..CG--........AGAG----------------------------------------...G..........................GCCGGG----..........................................----...............................................

Gua14b_A ...................-..CG--........AGAG----------------------------------------...G..........................GCCGGG----........................................------...............................................

Gua14a_D ...................-..CG--........AGAGAGAGAGAGAGAGAGAG------------------------...G........T.................G.CGGG----........................................------...............................................

Gua14b_D ...................-..CG--........AGAGAGAGAGAGAGAGAGAG------------------------...G........T.................G.CGGG----........................................------...............................................

Gua18a_B ...................-..CG--........AGAGAGAGAGAGAGAGAGAGAGAGAGAGAGAGAGAGAGAGAGAG...G........T.................G.CGGG----..........................................----...............................................

Gua18b_B ...................-..CG--........AGAGAGAGAGAGAGAGAGAGAGAGAGAGAGAGAGAGAGAGAGAG...G........T.................G.CGGG----..........................................----...............................................

Gua2a_bro ...................-..CG--........AGAGAGAGAGAGAGAGAGAGAGAG--------------------...G........T.................G.CGGG----..........................................----...............................................

Gua2b_bro ...................-..CG--........AGAGAGAGAGAGAGAGAGAGAGAG--------------------...G........T.................G.CGGG----..........................................----...............................................

Ita01a ...................A..----........AGAGAGAGAG----------------------------------...G..........................G.--------....................................----------...............................................

Ita01b ...................A..----........AGAGAGAGAG----------------------------------...G..........................G.--------....................................----------...............................................

Ita02a ...................A..----........AGAGAGAG------------------------------------...G..........T...............G.--------....................................----------...............................................

Ita02b ...................A..----........AGAGAGAGAG----------------------------------...G..........................G.--------....................................----------...............................................

Ita03a ...................A..----........AGAGAGAGAG----------------------------------...G..........................G.--------....................................----------...............................................

Ita03b ...................A..----........AGAGAGAG------------------------------------...G..........................G.--------....................................----------...............................................

Ita04a ...................A..----........AGAGAG--------------------------------------...G..........................G.--------....................................----------...............................................

Ita04b ...................A..----........AGAGAGAG------------------------------------...G..........................G.--------....................................----------...............................................

Ita05a ...................A..----........AGAG----------------------------------------...G..........................G.CGGG----....................................----------...............................................

Ita05b ...................A..----........AGAG----------------------------------------...G..........................G.CGGG----....................................----------...............................................

Ita06a ...................A..----........AGAG----------------------------------------...G..........................G.CGGG----....................................----------...............................................

Ita06b ...................A..----........AGAG----------------------------------------...G..........................G.CGGG----....................................----------...............................................

Ita07a ...................A..----........AGAG----------------------------------------...G..T..........T............G.CGAG----......................A.............----------...............................................

Ita07b ...................A..----........AGAG----------------------------------------...G..T..........T............G.CGAG----......................A.............----------...............................................

Ita08a ...................A..----........AGAGAGAG------------------------------------...G..........T...............G.--------....................................----------...............................................

Ita08b ...................A..----........AGAGAGAGAG----------------------------------...G..........................G.--------....................................----------...............................................

Ita09a ...................A..----........AGAG----------------------------------------...G..........................G.CGGC----......................A.............----------...............................................

Ita09b ...................A..----........AGAG----------------------------------------...G..........................G.CGGG----....................................----------...............................................

Ita10a ...................A..----........AGAGAGAG------------------------------------...G..........T...............G.--------....................................----------...............................................

Ita10b ...................A..----........AGAGAGAG------------------------------------...G..........................G.--------....................................----------...............................................

Ita11a ...................A..----........AGAGAGAG------------------------------------...G..........T...............G.--------....................................----------...............................................

Ita11b ...................A..----........AGAGAGAGAG----------------------------------...G..........................G.--------....................................----------...............................................

Ita12a ...................A..----........AGAG----------------------------------------...G..........................G.CGGC----......................A.............----------...............................................

Ita12b ...................A..----........AGAGAGAGAG----------------------------------...G..........................G.--------....................................----------...............................................

Ita14a ...................A..----........AGAGAGAG------------------------------------...G..........................G.--------....................................----------...............................................

Ita14b ...................A..----........AGAGAG--------------------------------------...G..........................G.--------....................................----------...............................................

Ita15a ...................A..----........AGAGAGAGAG----------------------------------...G..........................G.--------....................................----------...............................................

Ita15b ...................A..----........AGAGAGAG------------------------------------...G..........................G.--------....................................----------...............................................

Ita16a ...................A..----........AGAG----------------------------------------...G.............T............G.CGAG----......................A.............----------...............................................

Ita16b ...................A..----........AGAG----------------------------------------...G.............T............G.CGAG----......................A.............----------...............................................

Ita17a ...................A..----........AGAGAG--------------------------------------...G........................------------....................................----------...............................................

Ita17b ...................A..----........AGAGAG--------------------------------------...G........................------------....................................----------...............................................

Ita18a ...................A..----........AGAGAGAGAG----------------------------------...G..........................G.--------....................................----------...............................................

Ita18b ...................A..----........AGAGAGAGAG----------------------------------...G..........................G.--------....................................----------...............................................

Ita19a ...................A..----........AGAGAGAG------------------------------------...G..........................G.--------....................................----------...............................................

Ita19b ...................A..----........AGAGAGAG------------------------------------...G..........................G.--------....................................----------...............................................

Ita21a ...................A..----........AGAGAGAG------------------------------------...G..........................G.--------....................................----------...............................................

Ita21b ...................A..----........AGAGAGAG------------------------------------...G..........................G.--------....................................----------...............................................

Ita31a ...................A..----........AGAGAGAG------------------------------------...G.....G....................G.--------....................................----------...............................................

Ita31b ...................A..----........AGAGAGAG------------------------------------...G.....G....................G.--------..............................A.....----------...............................................

Ita41a ...................A..----........AGAGAG--------------------------------------...G........................------------....................................----------...............................................

Ita41b ...................A..----........AGAGAG--------------------------------------...G........................------------....................................----------...............................................

Sana01a ...................-..CG--........AGAG----------------------------------------...G..........................GCCGGG----..........................................----...............................................

Sana01b ...................-..CG--........AGAG----------------------------------------...G..........................GCCGGG----..........................................----...............................................

Sana02a ...................-..CGCG........AG------------------------------------------...G...............A..........G.CGGG----..........................................CT--...............................................

Sana02b ...................-..CGCG........AG------------------------------------------...G...............A..........G.CGGG----..........................................----...............................................

Sana04a ...................-..CGCG........AG------------------------------------------...G...............A..........G.CGGG----..........................................CT--...............................................

Sana04b ...................-..CGCG........AG------------------------------------------...G...............A..........G.CGGG----..........................................CT--...............................................

Sana43a ...................-..CG--........AGAGAG--------------------------------------...G..........................G.CGGG----..........................................----...............................................

Sana43b ...................-..CG--........AGAG----------------------------------------...G..........................G.CGGG----..........................................----...............................................

Sana751a ...................-..CG--........AGAG----------------------------------------...G..........................GCCGGG----..........................................----...............................................

Sana751b ...................-..CG--........AGAG----------------------------------------...G..........................GCCGGG----..........................................----...............................................

Sana752a ...................-..CG--........AGAGAGAGAGAGAG------------------------------...G..........................G.CGGG----..........................................----........A......................................

Sana752b ...................-..CG--........AGAGAGAGAGAG--------------------------------...G..........................G.CGGG----..........................................----........A......................................

Sana753a ...................-..CGCG........AG------------------------------------------...G...............A........T.G.CGGG----..........................................CT--...............................................

Sana753b ...................-..CGCG........AG------------------------------------------...G...............A........T.G.CGGG----..........................................CT--...............................................

Sana754a ...................-..CG--........AGAGAGAGAGAGAG------------------------------...G..........................G.CGGG----..........................................----........A......................................

Sana754b ...................-..CG--........AGAGAGAGAGAG--------------------------------...G..........................G.CGGG----..........................................----........A......................................

Sana756a ...................-..CG--........AGAGAGAGAGAGAG------------------------------...G..........................G.CGGG----..........................................----........A......................................

Sana756b ...................-..CG--........AGAGAGAGAGAG--------------------------------...G..........................G.CGGG----..........................................----........A......................................

Sana757a ...................-..CGCG........AG------------------------------------------...G...............A..........G.CGGG----..........................................CT--...............................................

Sana757b ...................-..CGCG........AG------------------------------------------...G...............A..........G.CGGG----..........................................CT--...............................................

Sana759a ...................-..CG--........AGAG----------------------------------------...G..........................GCCGGG----..........................................----...............................................

Sana759b ...................-..CG--........AGAG----------------------------------------...G..........................GCCGGG----..........................................----...............................................

Sana760a ...................-..CGCG........AG------------------------------------------...G...............A..........G.CGGG----..........................................CT--...............................................

Sana760b ...................-..CGCG........AG------------------------------------------...G...............A..........G.CGGG----..........................................CT--...............................................

Tin01a ...................-..CG--........AGAGAGAGAGAGAGAGAGAGAGAGAGAG----------------...G........T.................G.CGGG----..........................................----...............................................

Tin01b ...................-..CG--........AGAGAGAGAGAGAGAGAGAGAG----------------------...G........T.................G.CGGG----..........................................----...............................................

Tin02a ...................-..CG--........AGAG----------------------------------------...G..........................G-CGGGCGGG....................................T.....CT--...............................................

Tin02b ...................-..CG--........AGAG----------------------------------------...G..........................G-CGGGCGGG....................................T.....CT--...............................................

Tin03a ...................-..CG--........AGAG----------------------------------------...G..........................GCCGGG----........................................------...............................................

Tin03b ...................-..CG--........AGAG----------------------------------------...G..........................GCCGGG----..........................................----...............................................

Tin04a ...................-..CG--........AGAG----------------------------------------...G..........................GCCGGG----..........................................----...............................................

Tin04b ...................-..CG--........AGAG----------------------------------------...G..........................GCCGGG----..........................................----...............................................

Tin05a ...................-..CG--........AGAGAGAGAGAGAGAGAGAG------------------------...G........T.................G.CGGG----..........................................----...............................................

Tin05b ...................-..CG--........AGAGAGAGAGAGAGAGAG--------------------------...G........T.................G.CGGG----..........................................----...............................................

Tin06a ...................-..CG--........AGAGAGAGAGAGAGAGAGAG------------------------...G........T.................G.CGGG----..........................................----...............................................

Tin06b ...................-..CG--........AGAGAGAGAGAGAGAGAG--------------------------...G........T.................G.CGGG----..........................................----...............................................

Tin07a ...................-..CG--........AGAGAGAG------------------------------------...G..........................GCCGGG----..........................................----...............................................

Tin07b ...................-..CG--........AGAG----------------------------------------...G..........................GCCGGG----..........................................----...............................................

Tin09a ...................-..CG--........AGAG----------------------------------------...G..........................G.CGGG----..........................................----...............................................

Tin09b ...................-..CG--........AGAG----------------------------------------...G..........................G.CGGG----..........................................----...............................................

Tin10a ...................-..CG--........AGAG----------------------------------------...G..........................GCCGGG----..........................................----...............................................

Tin10b ...................-..CG--........AG------------------------------------------...G..........................GCCGGG----..........................................----...............................................

Tin11a ...................-..CG--........AGAA----------------------------------------...G..........................G.CGGG----..........................................----...............................................

Tin11b ...................-..CG--........AGAA----------------------------------------...G..........................G.CGGG----..........................................----...............................................

Alignment of the DNA sequences from the *Clock* gene fragment.

00000000000000000000000000000000000000000000000000000000000000000000000000000000000000000000000000011111111111111111111111111111111111111111111111111111111111111111111111111111111111111111111111111112222222222222222222222222

00000000011111111112222222222333333333344444444445555555555666666666677777777778888888888999999999900000000001111111111222222222233333333334444444444555555555566666666667777777777888888888899999999990000000000111111111122222

12345678901234567890123456789012345678901234567890123456789012345678901234567890123456789012345678901234567890123456789012345678901234567890123456789012345678901234567890123456789012345678901234567890123456789012345678901234

Boc05a GTGGCTTGTCATGAAGCATGTAAGCGCTACAATACTTCTACTCTTGTGATCTTCCCCCTGAGGGCTGTCAATGATTTAAA---------------------TCCGTTTCTTTTCTTCCGTT--GGATTTT-------------TGGGGTTCCCCTTCGCGTGC--------------------------TGCAGTGATGCAAAAGGGCGAGGGAACTTCGTGCT

Boc05b ................................................................................---------------------....................--.......-------------....................--------------------------...................................

Boc07a .........................A.........................--------------------........TA-------------ATTTAAA.............A......--..T....T------------G.C....T.T.CC.T.....GGTTTCTCCCCTCTCCCCTTTTCTTTG..................................

Boc07b ................................................................................---------------------....................--.......-------------....................--------------------------...................................

Boc08a .........................A.........................--------------------........TA-------------ATTTAAA.............A......--..T....T------------G.C....T.T.CC.T.....--------------------------...................................

Boc08b ......................................................A...........C.............---------------------..T..........A......--..------------------..C....T.T..C.....A.--------------------------...................................

Boc12a .........................A.........................--------------------........TA-------------ATCTAAA.............A......--..T....T------------G.C....T.T.CC.T.....--------------------------...................................

Boc12b .........................A.........................--------------------........TA-------------ATCTAAA.............A......--..T....T------------G.C....T.T.CC.T.....--------------------------...................................

Boc17a .........................A.........................--------------------........TA-------------ATCTAAA..T.................--..G....-------------.......T............--------------------------...................................

Boc17b .........................A...............G.........--------------------........TA-ATCTGATGAACGATTTAAA....................--..G....-------------.......T............--------------------------...................................

Boc18a .........................A...............G.........--------------------........TA-ATCTGATGAACGATTTAAA....................--..G....-------------.......T............--------------------------...................................

Boc18b .........................A...............G.........--------------------........TA-ATCTGATGAACGATTTAAA....................--..G....-------------.......T............--------------------------...................................

Boc19a .........................A.........................--------------------........TA-------------ATCTAAA..T.................--..G....-------------.......T............--------------------------...................................

Boc19b .........................A...............G.........--------------------........TA-ATCTGATGAACGATTTAAA....................--..G....-------------.......T............--------------------------...................................

Boc20a .........................A...............G.........--------------------........TA-ATCTGATGAACGATTTAAA....................--..G....-------------.......T............--------------------------...................................

Boc20b .........................A...............G.........--------------------........TA-ATCTGATGAACGATTTAAA....................--..G....-------------.......T............--------------------------...................................

Boc21a .........................A.........................--------------------........TA-------------ATCTAAA....................--..G....CCGTTCCGTTTT-.......T............--------------------------.....................A.............

Boc21b .........................A.........................--------------------........TA-------------ATCTAAA....................--..G....CCGTTCCGTTTT-.......T............--------------------------.....................A.............

Boc23a .........................A...............G.........--------------------........TA-ATCTGATGAACGATTTAAA....................--..G....-------------.......T............--------------------------...................................

Boc23b .........................A.........................--------------------........TA-------------ATCTAAA..T.................--..G....-------------.......T............--------------------------...................................

Boc24a .........................A...............G.........--------------------........TA-ATCTGATGAACGATTTAAA....................--..G....-------------.......T............--------------------------...................................

Boc24b .........................A...............G.........--------------------........TA-ATCTGATGAACGATTTAAA....................--..G....-------------.......T............--------------------------...................................

Boc25a .........................A...............G.........--------------------........TA-ATCTGATGAACGATTTAAA....................--..G....-------------.......T............--------------------------...................................

Boc25b .........................A...............G.........--------------------........TA-ATCTGATGAACGATTTAAA....................--..G....-------------.......T............--------------------------...................................

Boc26a .........................A...............G.........--------------------........TA-ATCTGATGAACGATTTAAA....................--..G....-------------.......T............--------------------------...................................

Boc26b .........................A.........................--------------------........TA-------------ATCTAAA..T.................--..G....-------------.......T............--------------------------...................................

Boc28a .........................A...............G.........--------------------........TA-ATCTGATGAACGATTTAAA....................--..G....-------------.......T............--------------------------...................................

Boc28b .........................A...............G.........--------------------........TA-ATCTGATGAACGATTTAAA....................--..G....-------------.......T............--------------------------...................................

Boc29a .........................A...............G.........--------------------........TA-ATCTGATGAACGATTTAAA....................--..G....-------------.......T............--------------------------...................................

Boc29b .........................A.........................--------------------........TA-------------ATCTAAA..T.................--..G....-------------.......T............--------------------------...................................

Boc30a .........................A........................T--------------------........TA----TGATGAATGATTTAAA....................--.......-------------G......T...G........--------------------------...................................

Boc30b .........................A.........................--------------------........TA-------------ATCTAAA....................--..G....-------------.......T............--------------------------...................................

Flo01a .................................................................GC.....A.......---------------------....................--..------------------.AC.C..T.T..C.......--------------------------...................................

Flo01b .................................................................GC.....A.......---------------------....................--..------------------.AC.C..T.T..C.......--------------------------...................................

Flo03a .................................-................................C.............---------------------....G...............--..------------------..C....T.T..C.......--------------------------...................................

Flo03b ..................................................................C.............---------------------....................--..------------------..C....T.T..C...C...--------------------------...................................

Flo04a ..................................................................C.............---------------------....G...............--..------------------..C....T.T..C.......--------------------------...................................

Flo04b ..................................................................C.............---------------------....................--..------------------..C....T.T..C...C...--------------------------...................................

Flo05a ..................................................................C.............---------------------....G...............--..------------------..C....T.T..C.......--------------------------...................................

Flo05b ..................................................................C.............---------------------....................--..------------------..C....T.T..C...C...--------------------------...................................

Flo06a ..................................................................C.............---------------------....................--..------------------..C....T.T..C...C...--------------------------...................................

Flo06b .................................................................GC.....A.......---------------------....................--..------------------.AC.C..T.T..C.......--------------------------...................................

Flo07a .................................................................GC.....A.......---------------------....................--..------------------.AC.C..T.T..C.......--------------------------...................................

Flo07b ..................................................................C.............---------------------....................--..------------------..C....T.T..C...C...--------------------------...................................

Flo08a .................................-...............................GC.............---------------------....................--..------------------..C....T.T..C.......--------------------------...................................

Flo08b ..................................................................C.............---------------------....G...............--..------------------..C....T.T..C.......--------------------------...................................

Flo09a .................................-................................C.............---------------------....G...............--..------------------..C....T.T..C.......--------------------------...................................

Flo09b .......................................................T..........C.T...........---------------------....G...............--..------------------..C....T.TT.C.......--------------------------...................................

Flo10a ..................................................................C.............---------------------....................--..------------------..C....T.T..C...C...--------------------------...................................

Flo10b ..................................................................C.............---------------------....................--..------------------..C....T.T..C...C...--------------------------...................................

Flo12a .................................-...............................GC.....A.......---------------------....................--..------------------.AC.C..T.T..C.......--------------------------...................................

Flo12b ..................................................................C.............---------------------....................--..------------------..C....T.T..C...C...--------------------------...................................

Flo16a .................................-................................C.............---------------------....G...............--..------------------..C....T.T..C.......--------------------------...................................

Flo16b ..................................T..............................GC.............--------------------T....G...............--..------------------..C....T.T..C.......--------------------------...................................

Flo20a ..................................................................C.............---------------------....................--..------------------..C....T.T..C...C...--------------------------...................................

Flo20b ..................................................................C.............---------------------....................--..------------------..C....T.T..C...C...--------------------------...................................

Gua01a .................................-............C...................C.............---------------------....................--..------------------..C....T.T..C.......--------------------------...................................

Gua01b .................................................................GC.....A.......---------------------....................--..------------------.AC.C..T.T..C.......--------------------------...................................

Gua02a .................................-................................C.............---------------------....G...............--..------------------..C....T.T..C.......--------------------------...................................

Gua02b ...........................................C......................C.............---------------------....G...............--..------------------..C....T.T..C.......--------------------------...................................

Gua03a .................................-................................C.............---------------------....G...............--..------------------..C....T.T..C.......--------------------------.....................A.............

Gua03b .................................-................................C.............---------------------....................--..------------------..C....T.T..C....AA.--------------------------...................................

Gua04a ..................................................................C.............---------------------....................--..------------------..C....T.T..C...C...--------------------------...................................

Gua04b ..................................................................C.............---------------------....................--..------------------..C....T.T..C...C...--------------------------...................................

Gua06a ...............................................................C..C.............---------------------....................--..------------------..C....T.T..C....C..--------------------------...................................

Gua06b .................................................................GC.....A.......---------------------....................--..------------------.AC.C..T.T..C.......--------------------------...................................

Gua10a_F ...............................................A..................C.............---------------------....G...............--..TG...-------------.......T............--------------------------.................................T.

Gua10b_F .................................................................GC.....A.......---------------------....................--..------------------.AC.C..T.T..C.......--------------------------...................................

Gua10a_H .....A...........................................................GC.....A.......---------------------....................--..------------------.AC.C..T.T..C.......--------------------------...................................

Gua10b_H ..................................................................C.....A.......---------------------....................--..------------------.AC.C..T.T..C.......--------------------------...................................

Gua14a_D .................................-................................C.............---------------------....G...............--..------------------..C....T.T..C.......--------------------------.....................A.............

Gua14b_D ..................................................................C.............---------------------....................--..------------------..C....T.T..C.......--------------------------...................................

Gua18a_B .................................TG..............................GC.............---------------------...........C........--..------------------..C....T.T..C...C...--------------------------...................................

Gua18b_B .................................................................GC.....A.......---------------------..................A.--..------------------.AC.C..T.T..C.......--------------------------...................................

Ita02a .........................A.........................--------------------........TA-------------ATCTAAA....................--..G....-------------.......T............--------------------------...................................

Ita02b .........................A.........................--------------------........TA-------------ATCTAAA....................--..G....-------------.......T............--------------------------...................................

Ita03a .........................A.........................--------------------........TA-------------ATCTAAA..T.................--..G....-------------.......T............--------------------------...................................

Ita03b .........................A.........................--------------------........TA-------------ATCTAAA....................--..G....-------------.......T............--------------------------...................................

Ita04a .........................A.........................--------------------........TA-------------ATCTAAA.............A......--.......-------------G......T...G........--------------------------...................................

Ita04b .........................A.........................--------------------........TA-------------ATCTAAA..T.................--..G....-------------.......T............--------------------------...................................

Ita05a .........................A.........................--------------------........TG-ATCTGATGAATGATTTAAA....................--..G....-------------.......T............--------------------------...................................

Ita05b .........................A.........................--------------------........TA-------------ATTTAAA.............A......--..T....T------------G.C....T.T.CC.T.....--------------------------...................................

Ita06a .........................A.........................--------------------........TA-------------ATCTAAA....................--..G....ACGTTCCGTTTT-.......T............--------------------------...................................

Ita06b .........................A.........................--------------------........TA-ATCTGATGAATGATTTAAA....................--..-....-------------.......T.A.CC.T.....--------------------------...................................

Ita07a .........................A.........................--------------------........TA-------------ATCTAAA....................--..G....CCGTTCCGTTTT-.......T............--------------------------.....................A.............

Ita07b .........................A........................T--------------------........TA----TGATGAATGATTTAAA....................--.......-------------G......T...G........--------------------------...................................

Ita08a .........................A.........................--------------------........TA-------------ATCTAAA....................--..G....-------------.......T............--------------------------...................................

Ita08b .........................A.........................--------------------........TA-------------ATCTAAA..T.................--..G....-------------.......T............--------------------------...................................

Ita09a .........................A.........................--------------------........TA-------------ATCTAAA.............A......--..T....T------------G.C....T.T.CC.T.....--------------------------...................................

Ita09b .........................A.........................--------------------........TA-------------ATCTAAA....................--..G....CCGTTCCGTTTT-.......T............--------------------------.....................A.............

Ita10a .........................A.........................--------------------........TA-------------ATCTAAA....................--..G....-------------.......T............--------------------------...................................

Ita10b ...................................................--------------------........TA-------------ATCTAAA....................--.......-------------.......T........A...--------------------------...................................

Ita11a .........................A.........................--------------------........TA-------------ATCTAAA....................--..G....-------------.......T............--------------------------...................................

Ita11b ...................................................--------------------........TA-------------ATCTAAA....................--.......-------------.......T........A...--------------------------...................................

Ita12a .........................A.........................--------------------........TA-------------ATCTAAA....................--..G....-------------.......T............--------------------------...................................

Ita12b .........................A.........................--------------------........TA-------------ATCTAAA..T.................--..G....-------------.......T............--------------------------...................................

Ita13a .........................A.........................--------------------........TA-------------ATCTAAA..T.................--..G....-------------.......T............--------------------------...................................

Ita13b .........................A.........................--------------------........TG-ATCTGATGAATGATTTAAA..G.................--..G....-------------.......T............--------------------------...................................

Ita14a .........................A.........................--------------------........TA-------------ATCTAAA....................--..G....-------------.......T............--------------------------...................................

Ita14b .........................A.........................--------------------........TA-------------ATCTAAA....................--..G....-------------.......T............--------------------------...................................

Ita15a .........................A.........................--------------------........TA-------------ATCTAAA....................--..G....-------------.......T............--------------------------...................................

Ita15b ...................................................--------------------........TA-------------ATCTAAA....................--.......-------------.......T........A...--------------------------...................................

Ita16a .........................A...............G.........--------------------........TA-ATCTGATGAACGATTTAAA....................--..G....-------------.......T............--------------------------...................................

Ita16b .......................................T...........--------------------........TG-ATCTGATGAATGATTTAAA....................CC...A...-------------.......T............--------------------------...................................

Ita17a .........................A...............G.........--------------------........TA-ATCTGATGAACGATTTAAA....................--..G....-------------.......T............--------------------------...................................

Ita17b .........................A...............G.........--------------------........TA-ATCTGATGAACGATTTAAA....................--..G....-------------.......T............--------------------------...................................

Ita18a .........................A...............G.........--------------------........TA-ATCTGATGAACGATTTAAA....................--..G....-------------.......T............--------------------------...................................

Ita18b .........................A...............G.........--------------------........TA-ATCTGATGAACGATTTAAA....................--..G....-------------.......T............--------------------------...................................

Ita19a .........................A.........................--------------------........TA-------------ATCTAAA..T.................--..G....-------------.......T............--------------------------...................................

Ita19b .........................A...............G.........--------------------........TA-ATCTGATGAACGATTTAAA....................--..G....-------------.......T............--------------------------...................................

Ita21a .........................A...............G.........--------------------........TA-ATCTGATGAACGATTTAAA....................--..G....-------------.......T............--------------------------...................................

Ita21b .........................A...............G.........--------------------........TA-ATCTGATGAACGATTTAAA....................--..G....-------------.......T............--------------------------...................................

Ita31a .........................A...............G.........--------------------........TA-ATCTGATGAACGATTTAAA....................--..G....-------------.......T............--------------------------...................................

Ita31b .........................A...............G.........--------------------........TA-ATCTGATGAACGATTTAAA....................--..G....-------------.......T............--------------------------...................................

San01a .................................-................................C.............---------------------....G...............--..------------------..C....T.T..C.......--------------------------.....................A.............

San01b .................................-................T...............C.............---------------------....................--..------------------..C....T.T..C.......--------------------------...................................

San02a ..................................................................C.............---------------------....................--..------------------..C....T.T..C.......--------------------------...................................

San02b .................................-................................C.............---------------------....G...............--..------------------..C....T.T..C.......--------------------------.....................A.............

San04a ..................................................................C.............---------------------....................--..------------------..C....T.T..C.......--------------------------...................................

San04b ..................................................................C.............---------------------....................--..------------------..C....T.T..C.......--------------------------...................................

San05a ..................................................................C.............---------------------....................--..------------------..C....T.T..C.......--------------------------...................................

San05b ..................................................................C.............---------------------....................--..------------------..C....T.T..C.......--------------------------...................................

San06a .................................-...................T............C..........T..---------------------...A................--...C.G.-------------..T....-.T.CC..T....--------------------------...................................

San06b ..................................................................C.............---------------------....................--..------------------..C....T.T..C.......--------------------------...................................

San07a ..................................................................C.............---------------------....................--..------------------..C....T.T..C.......--------------------------...................................

San07b ..................................................................C.............---------------------....................--..------------------..C....T.T..C.......--------------------------...................................

San40a ......................................................A...........C.............---------------------..T..........A......--..------------------..C....T.T..C.......--------------------------...................................

San40b ..................................................................C..........T..---------------------....................--..------------------..C....TTT..C...C...--------------------------...................................

San42a ..................................................................C.............---------------------....................--..------------------..C....T.T..C.......--------------------------...................................

San42b ..................................................................C.............---------------------....G...............--..------------------..C....T.T..C.......--------------------------...................................

San43a .................................-................................C.............---------------------....G...............--..------------------..C....T.T..C.......--------------------------.....................A.............

San43b .................................-................T...............C.............---------------------....................--..------------------..C....T.T..C.......--------------------------...................................

Tin03a .................................................................GC.....A.......---------------------....................--..------------------.AC.C..T.T..C.......--------------------------...................................

Tin03b .................................................................GC.....A.......---------------------....................--..------------------.AC.C..T.T..C.......--------------------------...................................

Tin04a_J ..................................................................C.............---------------------....................--..------------------..C....T.T..C.......--------------------------.................................T.

Tin04b_J ..................................................................C.............---------------------....G..A............--..TG...-------------.......T............--------------------------.................................T.

Tin05a_N ..................................................................C..G..........---------------------....G...............--..------------------..C....T.T..C.......--------------------------...................................

Tin05b_N .................................-....................A...........C.............---------------------..T..........A......--..------------------..C....T.T..C.......--------------------------...................................

Tin05a_Q ..................................................................C..........T..---------------------....................--..------------------..C....TTT..C...C...--------------------------...................................

Tin05b_Q ......................................................A...........C.............---------------------..T..........A......--..------------------..C....T.T..C.......--------------------------...................................

Tin05a_T .................................-....................A...........C.............---------------------..T..........A......--..------------------..C....T.T..C.......--------------------------...................................

Tin05b_T ..................................................................C..G..........---------------------....G...............--..------------------..C....T.T..C.......--------------------------...................................

Tin09a_A ..................................................................CA.........T..---------------------....................--..------------------..C....TTT..C...C...--------------------------...................................

Tin09b_A ..................................................................C..........G..---------------------....................--..------------------..C....T.T..C.......--------------------------...................................

Tin09a_T .................................-................................C.............---------------------....................--..------------------..C....T.T..C.......--------------------------...................................

Tin09b_T ..................................................................C..........G..---------------------....................--..------------------..C....T.T..C.......--------------------------...................................

Tin20a_C .................................-............C...................C.............---------------------....................--..------------------..C....T.T..C.......--------------------------...................................

Tin20b_C .................................TG...............................C.............---------------------....G...............--..------------------..C....T.T..C.......--------------------------...................................

Tin20a_H ..................................................................C.....A.......---------------------....................--..------------------.AC.C..T.T..C.......--------------------------...................................

Tin20b_H .................................-................................C.............---------------------....G...............--..------------------..C....T.T..C...C...--------------------------...................................

Tin20a_P .................................-....................T.......C...C.............---------------------....................--..------------------..C....T.T..C...C...--------------------------...................................

Tin20b_P ..................................................................C..........T..---------------------....................--..------------------..C....TTT..C...C...--------------------------...................................
